# Mesenchymal stem cells alleviate systemic sclerosis by inhibiting the recruitment of pathogenic macrophages

**DOI:** 10.1038/s41420-022-01264-2

**Published:** 2022-11-26

**Authors:** Pixia Gong, Yayun Ding, Rongrong Sun, Zishan Jiang, Wen Li, Xiao Su, Ruifeng Tian, Yipeng Zhou, Tingting Wang, Junjie Jiang, Peishan Li, Changshun Shao, Yufang Shi

**Affiliations:** The First Affiliated Hospital of Soochow University, State Key Laboratory of Radiation Medicine and Protection, Institutes for Translational Medicine, Suzhou Medical College of Soochow University, Suzhou, Jiangsu 215123 China

**Keywords:** Mesenchymal stem cells, Autoimmunity

## Abstract

Systemic sclerosis (SSc) is a recalcitrant autoimmune disease for which there is no cure. Mesenchymal stem cell (MSC)-based treatment has emerged as a promising therapeutic option for several autoimmune diseases. Previously, we found that the immunoregulatory potential of MSCs can be greatly enhanced by IFN-γ and TNF-α. Here, we found that IFN-γ- and TNF-α-pretreated MSCs significantly alleviated skin fibrosis in a bleomycin (BLM)-induced SSc model. Macrophages were found to be the predominant profibrotic immune cell population in the pathogenesis of SSc. The accumulation of macrophages was significantly decreased by MSC treatment. Importantly, MSCs primarily reduced the population of maturing macrophages with high CCR2 expression by inhibiting the generation of CCL2 from fibroblasts and macrophages. This finding may help to improve MSC-based clinical treatments for SSc patients.

## Introduction

Systemic sclerosis (SSc), also known as scleroderma, is an autoimmune connective tissue disease characterized by progressive organ fibrosis, especially affecting the skin [1]. Aberrant immune responses have been recognized as prominent factors contributing to the pathogenesis of SSc. Monocytes [2], macrophages [3–5], dendritic cells [6], mast cells [7], and T cells [8] were found to accumulate in the skin of SSc patients. Moreover, T cells and macrophages exhibit an activated phenotype, indicating their critical roles in SSc [9, 10]. In addition, B-cell activation in SSc is associated with autoantibody production [11]. The extensive involvement of immune cells in SSc suggests that immune cells might be targeted for therapeutic interventions. However, recent microarray studies revealed diverse immune responses among SSc patients [12], which makes immune cell-targeted therapeutic regimens difficult. Immunosuppressive drugs, such as cyclophosphamide, only have modest effects without survival improvement [13]. Immunotherapies that target specific immune cell populations or cytokines have yet to be proven beneficial [14].

Mesenchymal stem cells (MSCs) have been emerging as a potential therapeutic option for SSc patients owing to their potent and extensive immunomodulatory capability. MSCs can inhibit the activation, proliferation, differentiation, maturation, and function of both innate and adaptive immune cells through secreted factors and molecules [15, 16]. It has been demonstrated that human umbilical cord-derived MSCs inhibit the infiltration and activation of T helper (Th) 17 cells in the skin in a bleomycin (BLM)-induced SSc model [17]. Bone marrow-derived MSCs impede the infiltration of T cells and macrophages into skin in a hypochlorite-induced SSc model [18]. Another study demonstrated that bone marrow-derived MSCs reduced the accumulation of macrophages and neutrophils but not T cells in the skin [19]. Nevertheless, the mechanism by which MSCs modulate the infiltration and activation of dysregulated immune systems in SSc is not well understood.

The types and levels of inflammation frequently affect the therapeutic effects of MSCs because the immunomodulatory capabilities of MSCs are not constitutive but rather are elicited by inflammatory cytokines [20]. Our group has demonstrated that the immunomodulatory capabilities of MSCs can be greatly augmented by interferon (IFN)-γ and tumor necrosis factor (TNF)-α (IT) pretreatment and thus achieve stable and reproducible therapeutic effects. The potential therapeutic effect of MSC-IT treatment has been proven in a lipopolysaccharide-induced acute lung injury model [21] and a skin wound healing model [22]. Therefore, MSCs-IT may be a better therapeutic option for SSc patients with heterogeneous inflammation states.

In the present study, we investigated the therapeutic effects and underlying mechanism of MSCs-IT in a BLM-induced SSc model. We found that MSCs-IT significantly alleviated skin fibrosis during SSc by reducing the infiltration of pathogenic CCR2^hi^ macrophages. MSC-IT treatment reduced the generation of CCL2 from fibroblasts and macrophages to inhibit macrophage recruitment. Our results provide insight into the mechanism underlying MSC efficacies in autoimmune diseases.

## Results

### MSC-IT treatment alleviates BLM-induced SSc

**Fig. 1:**
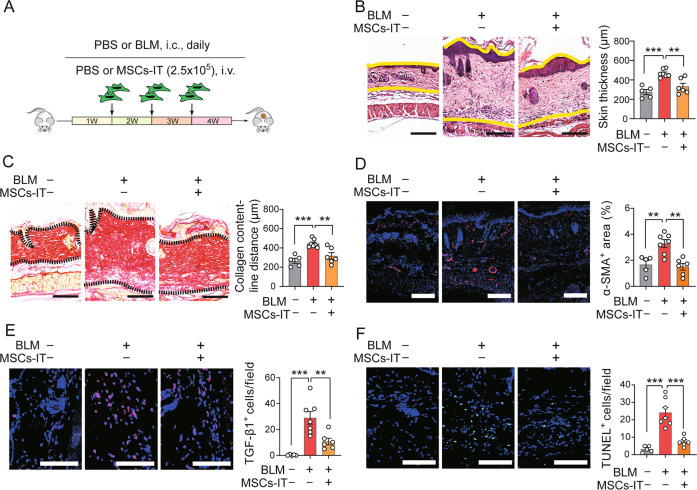
MSC-IT treatment alleviates BLM-induced SSc. **A** Experimental design of the BLM-induced SSc mouse model and MSC-IT treatment. Mice were i.c. injected with BLM or PBS daily for 4 weeks. MSCs were pretreated with 10 ng/mL IFN-γ and 10 ng/mL TNF-α for 24 h, and then MSCs-IT (2 × 10^5^) were i.v. injected into mice on days 7, 14, and 21. Finally, mice were sacrificed and examined on day 30. *n* = 5–7 mice for each group. **B** H&E staining of the skin sections. The distance between the indicated yellow lines was assessed. Scale bars, 200 μm. **C** Sirius Red staining of the skin sections. The distance between the indicated two black dotted lines was assessed. Scale bars, 200 μm. **D** Immunofluorescence staining of α-SMA (red) in the skin sections. The percentage of α-SMA^+^ area was assessed. Scale bars, 200 μm. **E** Immunofluorescence staining of TGF-β1 (red) in skin sections. The number of TGF-β1^+^ cells per field was counted. Scale bars, 50 μm. **F** TUNEL apoptosis assay of the skin sections. The green fluorescence represents apoptotic cells and the number of TUNEL-positive cells per field was counted. Scale bars, 50 μm. Data were shown as means ± SEM. Data are representative of three experiments with similar results. **P < 0.01; ***P < 0.001.

To investigate the effects of MSCs-IT on skin fibrosis during SSc, a BLM-induced SSc mouse model was established and treated with MSC-IT via intravenous injection (Fig. 1A). MSC-IT treatment significantly ameliorated skin thickness and collagen deposition in SSc mice, as shown by H&E staining and Sirius Red staining (Fig. 1B, C). We further found that the expression of α-SMA, a fibrosis marker, was significantly reduced after MSC-IT treatment (Fig. 1D). The expression of TGF-β1, a profibrotic factor in SSc [23], was also reduced in the MSC-IT-treated group (Fig. 1E). Moreover, cells in the skin were more resistant to apoptosis in the MSC-IT treatment group, as demonstrated by TUNEL staining (Fig. 1F). Collectively, these results indicate that MSCs-IT are capable of mitigating the development of skin fibrosis during SSc.

### Myeloid cells play a key role in the pathogenesis of SSc

**Fig. 2:**
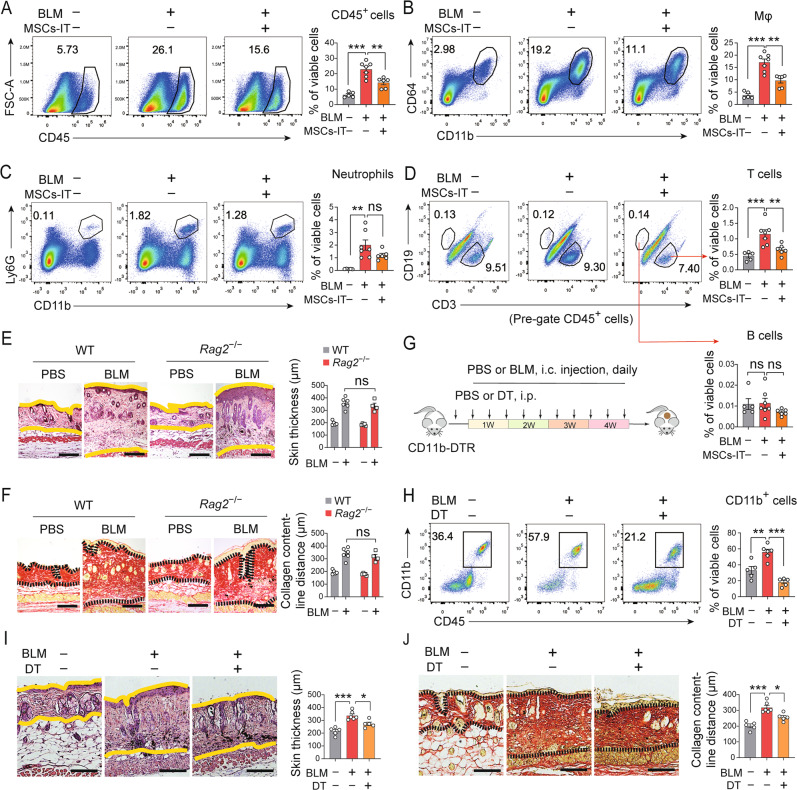
Myeloid cells play a key role in the pathogenesis of SSc. **A–D** Mice were i.c. injected with BLM or PBS daily for 4 weeks. MSCs-IT (2 × 10^5^) were i.v. injected into SSc mice on days 7, 14, and 21. Mice were sacrificed and subjected to flow cytometry analysis on day 30. *n* = 5–7 mice for each group. **A** Flow cytometry analysis of CD45^+^ immune cells in the skin. **B** Flow cytometry analysis of CD11b^+^CD64^+^ macrophages in the skin. **C** Flow cytometry analysis of CD11b^+^Ly6G^+^ neutrophils in the skin. **D** Flow cytometry analysis of CD3^+^ T cells and CD19^+^ B cells in the skin. **E**, **F** *Rag 2*^−/−^ mice and WT mice were i.c. injected with BLM or PBS daily for 4 weeks. Mice were sacrificed and examined on day 30. *n* = 4–6 mice for each group. **E** H&E staining of skin sections from *Rag 2*^−/−^ mice and WT mice. The distance between the two yellow lines was assessed. Scale bars, 200 μm. **F** Sirius Red staining of skin sections from *Rag 2*^−/−^ mice and WT mice. The distance between two black dotted lines was assessed. Scale bars, 200 μm. **G** Experimental design of the BLM-induced SSc model of CD11b-DTR mice and DT treatment. Mice were sacrificed and examined on day 30. *n* = 5–6 mice for each group. **H** Depletion efficiency of DT treatment on CD11b^+^ myeloid cells in the skin of CD11b-DTR mice was analyzed by flow cytometry on day 30. **I** H&E staining of skin sections from CD11b-DTR mice. The distance between the two yellow lines was assessed. Scale bars, 200 μm. **J** Sirius Red staining of skin sections from CD11b-DTR mice. The distance between two black dotted lines was assessed. Scale bars, 200 μm. Data were shown as means ± SEM. Data are representative of two or three experiments with similar results. *P < 0.05; **P < 0.01; ***P < 0.001; ns not significant.

The fibrotic response in SSc is closely related to abnormalities in the immune system. Both innate and adaptive immune cells are reported to contribute to the pathogenesis of SSc [24]. To investigate the mechanism by which MSCs-IT alleviate SSc, we examined the effects of MSCs-IT on the infiltrated immune cells in the skin. MSC-IT treatment significantly hindered the infiltration of CD45^+^ immune cells into the skin lesion (Fig. 2A). In myeloid immune cells, macrophages were significantly reduced after MSC-IT treatment (Fig. 2B), while neutrophils were not obviously changed (Fig. 2C). In adaptive immune cells, T cells were the significantly decreased population (Fig. 2D, top), rather than B cells (Fig. 2D, bottom) after MSC-IT treatment. These results suggested that MSCs-IT mainly affected the infiltration of macrophages and T cells.

To investigate the role of T cells and B cells in the pathogenesis of SSc, *Rag2*^−/−^ mice, which lack T cells and B cells, were employed. H&E staining and Sirius Red staining showed that the skin thickness and collagen deposition were not significantly different between the WT mice and *Rag*2^−/−^ mice (Fig. 2E, F).

To investigate the role of myeloid immune cells in the pathogenesis of SSc, we employed CD11b-DTR mice, in which CD11b^+^ myeloid cells, including macrophages, monocytes, neutrophils, and dendritic cells, can be depleted by diphtheria toxin (DT) administration. DT was administered intraperitoneally every 4 days to BLM-treated mice starting 3 days before SSc induction to deplete CD11b^+^ myeloid cells (Fig. 2G). DT effectively depleted CD11b^+^ myeloid cells in the skin (Fig. 2H). H&E staining and Sirius Red staining showed that the skin thickness and collagen deposition were significantly decreased in the DT treatment group compared with the untreated group (Fig. 2I, J). These results suggested a more important role of myeloid cells than T and B cells in the BLM-induced SSc model. It can be inferred from the above results that macrophages, the most decreased cell population among myeloid cells after MSC-IT treatment, might determine the therapeutic effects of MSCs-IT.

### MSCs-IT ameliorate SSc by acting on macrophages

**Fig. 3:**
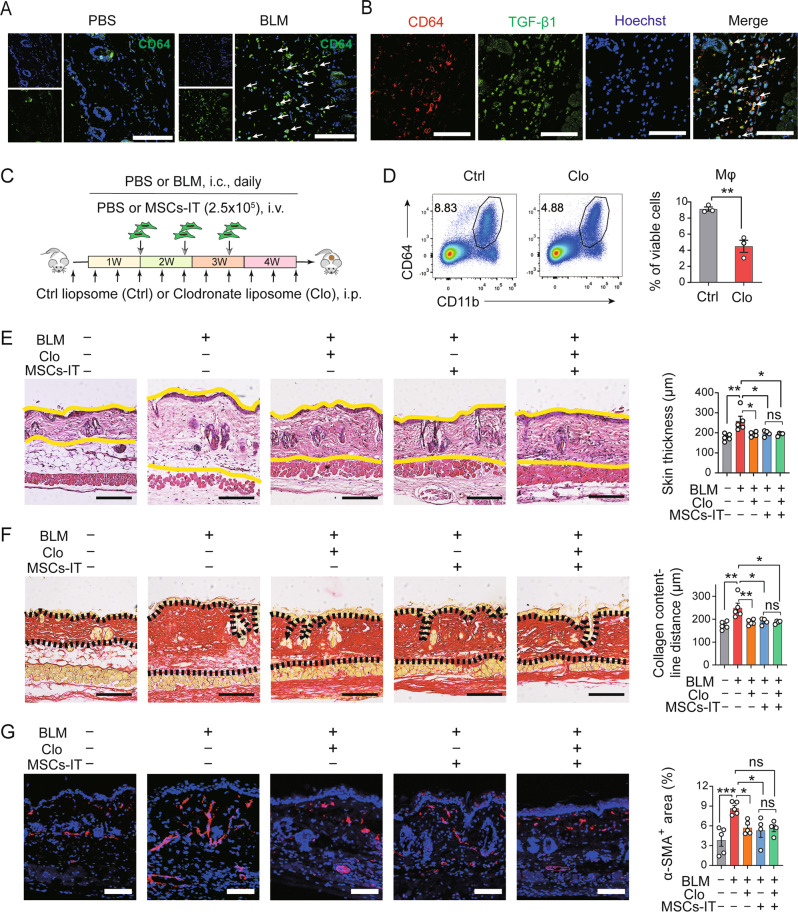
MSCs-IT ameliorate SSc by acting on macrophages. **A**, **B** Mice were i.c. injected with BLM or PBS daily for 4 weeks to induce SSc and then euthanized and examined on day 30. **A** The infiltration of macrophages in the skin was assessed by immunofluorescence staining of CD64 (green). Scale bars, 100 μm. **B** The colocalization of CD64 (red) and TGF-β1 (green) in the skin of SSc mice was determined by immunofluorescence staining. Scale bars, 100 μm. **C** Experimental design of the BLM-induced SSc mouse model, Clodronate liposome (Clo)/Control liposome (Ctrl) treatment, and MSC-IT treatment. Mice were euthanized and examined on day 30. *n* = 4–5 mice for each group. **D** 100 μL Clo liposomes (5 mg/mL) or 100 μL of Ctrl liposomes was administered to normal mice, and the depletion efficiency of Clo liposome treatment on macrophages (CD11b^+^CD64^+^) in the skin was analyzed by flow cytometry on day 3. *n* = 3 mice for each group. **E** H&E staining of the skin sections. The distance between the two yellow lines was assessed. Scale bars, 200 μm. **F** Sirius Red staining of the skin sections. The distance between two black dotted lines was assessed. Scale bars, 200 μm. **G** Immunofluorescence staining of α-SMA (red) in the skin sections. The percentage of α-SMA^+^ area was assessed. Scale bars, 200 μm. Data were shown as means ± SEM. Data are representative of two or three experiments with similar results. *P < 0.05; **P < 0.01; ***P < 0.001; ns not significant.

Macrophages substantially infiltrated into the dermal lesions during SSc, as shown by immunofluorescence staining of anti-CD64 antibody (Fig. 3A), and served as the predominant constituent of the skin immune cell pool. Further immunofluorescence analysis showed that macrophages were highly colocalized with the profibrotic factor TGF-β1, suggesting their potential profibrotic capability in SSc (Fig. 3B).

To examine whether the therapeutic effect of MSCs-IT is dependent on modulating macrophages, we employed clodronate liposomes (Clo) to deplete macrophages. In the process of SSc mouse model establishment, clodronate liposomes were administered intraperitoneally every 4 days to BLM-treated mice starting 3 days before model induction. MSCs-IT were injected intravenously on days 7, 14, and 21 (Fig. 3C). Clo liposomes effectively depleted macrophages in the skin (Fig. 3D). H&E staining and Sirius Red staining showed that Clo liposomes treatment significantly decreased skin thickness and collagen deposition (Fig. 3E, F, lane 3), confirming the profibrotic role of macrophages in SSc. Importantly, MSC-IT treatment exhibited no obvious therapeutic efficacy after macrophage depletion (Fig. 3E, F, lane 5 versus lane 4). Immunofluorescence staining of α-SMA also demonstrated no significant difference between the Clo liposomes alone group and the Clo liposomes and MSCs-IT combination group (Fig. 3G), indicating that MSCs-IT no longer have a beneficial effect on SSc when macrophages are depleted. Taken together, these results indicate that MSCs-IT alleviate BLM-induced SSc by acting on macrophages.

### MSCs-IT reduce the accumulation of CCR2^hi^-maturing macrophages

**Fig. 4:**
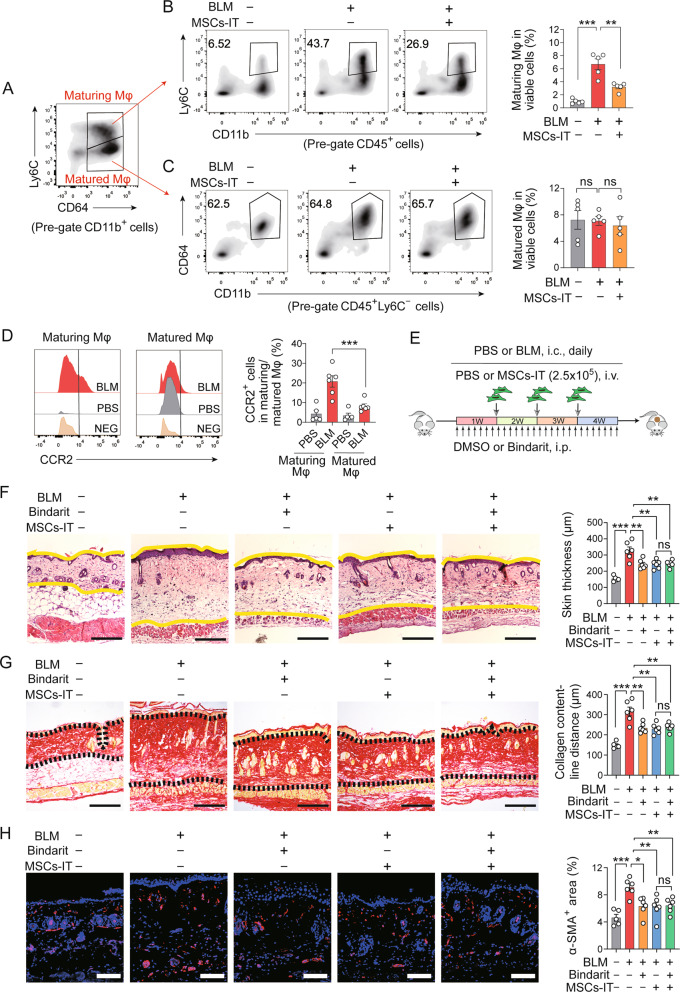
MSCs-IT mainly reduce CCR2^hi^-maturing macrophages. **A** The subpopulations of CD11b^+^CD64^+^ macrophages in the skin of SSc mice were analyzed and classified by flow cytometry according to the expression of Ly6C. **B** Flow cytometry analysis of CD11b^+^ (CD64^+^) Ly6C^+^ macrophages in MSCs-IT-treated or untreated mice on day 30. *n* = 5 mice for each group. **C** Flow cytometry analysis of CD11b^+^CD64^+^Ly6C^-^ macrophages in MSCs-IT-treated or untreated mice on day 30. *n* = 5 mice for each group. **D** CCR2 expression among CD64^+^Ly6C^+^ macrophages and CD64^+^Ly6C^-^ macrophages from BLM-treated or untreated mice was analyzed by flow cytometry. *n* = 5–6 mice for each group. NEG, negative. **E** Experimental design of the BLM-induced SSc mouse model, Bindarit treatment, and MSC-IT treatment. Mice were euthanized and examined on day 30. *n* = 5–6 mice for each group. **F** H&E staining of the skin sections. The distance between the two yellow lines was assessed. Scale bars, 200 μm. **G** Sirius Red staining of the skin sections. The distance between two black dotted lines was assessed. Scale bars, 200 μm. **H** Immunofluorescence staining of α-SMA (red) in the skin sections. The percentage of α-SMA^+^ area was assessed. Scale bars, 200 μm. Data were shown as means ± SEM. Data are representative of two or three experiments with similar results. *P < 0.05; **P < 0.01; ***P < 0.001; ns not significant.

Macrophages are composed of a group of heterogeneous cells. To investigate the potential subpopulation modulated by MSCs-IT in BLM-induced SSc, we divided CD11b^+^CD64^+^ macrophages into 2 subpopulations based on the expression of Ly6C, a monocyte marker (Fig. 4A). The CD64^+^Ly6C^+^ subpopulation was regarded as maturing macrophages representing the intermediate between monocytes and macrophages. CD64^+^Ly6C^-^ subpopulation represents matured macrophages. Flow cytometry analysis showed that CD64^+^Ly6C^+^ maturing macrophages significantly decreased in fibrotic skin after MSC-IT treatment (Fig. 4B). In contrast, MSC-IT treatment had no obvious influence on CD64^+^Ly6C^-^ matured macrophages (Fig. 4C), suggesting that the major target of MSCs-IT is CD64^+^Ly6C^+^ maturing macrophages, rather than CD64^+^Ly6C^-^ matured macrophages.

The CCL2-CCR2 chemokine-chemokine receptor axis mediates the migration of monocytes toward injury sites. We found that CD64^+^Ly6C^+^ maturing macrophages expressed more CCR2 than CD64^+^Ly6C^-^ matured macrophages (Fig. 4D), indicating that CD64^+^Ly6C^+^ maturing macrophages are transformed from the recruited CCR2^+^ monocytes. Thus, we speculated that MSC-IT treatment reduced CD64^+^Ly6C^+^ maturing macrophages by hindering the recruitment of peripheral monocytes. To test this hypothesis, the CCR2 inhibitor Bindarit was used in the process of SSc induction. Bindarit was administered intraperitoneally to BLM-treated mice daily starting from day 0, and MSCs-IT were injected intravenously on days 7, 14, and 21 (Fig. 4E). Bindarit treatment significantly decreased skin thickness and collagen deposition, as indicated by H&E staining and Sirius Red staining (Fig. 4F, G, lane 3). Further analysis showed that MSC-IT treatment had no further therapeutic effect on BLM-induced SSc after CCR2 inhibition (Fig. 4F, G, lane 5 versus lane 4). MSC-IT treatment also failed to further reduce α-SMA expression after CCR2 inhibition (Fig. 4H). These results indicate that MSCs-IT alleviate BLM-induced SSc by inhibiting the infiltration of monocytes.

### MSCs-IT inhibit CCL2 production by fibroblasts and macrophages

**Fig. 5:**
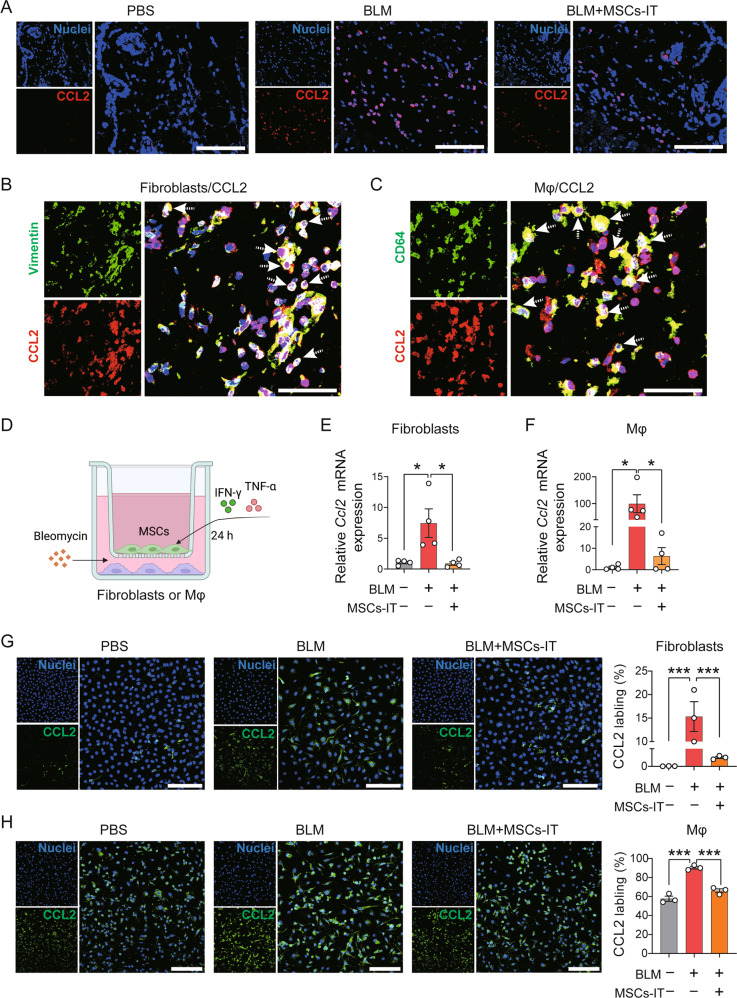
MSCs-IT inhibit CCL2 production by macrophages and fibroblasts. **A** Immunofluorescence staining of CCL2 (red) in skin sections from MSCs-IT treated or untreated mice on day 30. Scale bars, 100 μm. **B** Skin sections were stained with anti-Vimentin (green) and anti-CCL2 (red) antibodies to visualize the colocalization of fibroblasts and CCL2 in BLM-treated mice on day 30. White dashed arrows indicate the double-positive cells. Scale bars, 35 μm. **C** Skin sections were stained with anti-CD64 (green) and anti-CCL2 (red) antibodies to visualize the colocalization of macrophages and CCL2 in BLM-treated mice on day 30. White dashed arrows indicate the double-positive cells. Scale bars, 35 μm. **D** MSCs-IT were stimulated with 10 ng/mL IFN-γ and 10 ng/mL TNF-α in the inserts of transwells for 24 h, and then the inserts bearing MSCs-IT were placed on the top of multiwell plates adhering fibroblasts or macrophages in the presence of 10 ng/mL BLM. Then, the expression of CCL2 in fibroblasts or macrophages was examined at 24 h. **E** The expression of *Ccl2* mRNA in fibroblasts was examined by qPCR. *n* = 4. **F** The expression of *Ccl2* mRNA in macrophages was examined by qPCR. *n* = 4. **G** Fibroblasts were stained with anti-CCL2 (green) antibody to visualize the expression of CCL2. Scale bars, 100 μm. *n* = 3. **H** Macrophages were stained with anti-CCL2 (green) antibody to visualize the expression of CCL2. Scale bars, 100 μm. *n* = 3. Data were shown as means ± SEM. Data are representative of three experiments with similar results. *P < 0.05; ***P < 0.001.

To examine how MSCs-IT regulate the recruitment of monocytes, the expression of CCL2, a key ligand of CCR2, in the skin was determined by immunofluorescence staining. CCL2 was highly expressed in fibrotic skin (Fig. 5A, middle) but significantly decreased after MSC-IT treatment (Fig. 5A, right). Further immunofluorescence analysis revealed that fibroblasts and macrophages were the major cell populations generating CCL2 during SSc, as shown by the colocalization of CCL2 with Vimentin (marker of fibroblasts) or CD64 (marker of macrophages) (Fig. 5B, C).

**Fig. 6:**
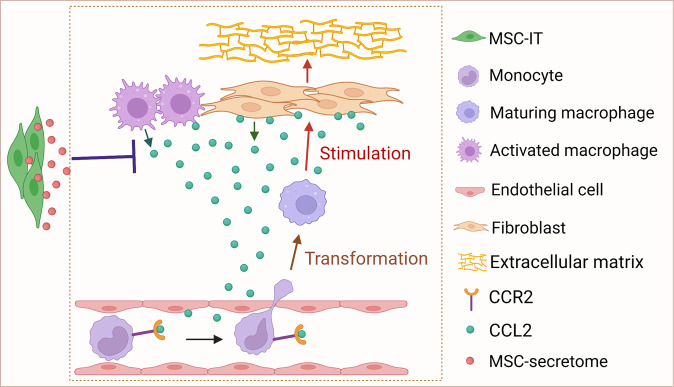
A diagrammatic model of the mechanism by which MSCs-IT regulate macrophages in BLM-induced SSc. Fibroblasts and macrophages generated substantial CCL2 during BLM-induced SSc to attract peripheral monocytes into skin lesions. These monocytes transform into CCR2^hi^-maturing macrophages, which can stimulate the production of extracellular matrix from fibroblasts and promote skin fibrosis. The infused MSCs-IT inhibited CCL2 generation from fibroblasts and macrophages, thus alleviating BLM-induced SSc.

To examine the effect of MSCs-IT on CCL2 generation, we co-cultured MSCs-IT with mouse skin-derived fibroblasts or bone marrow-derived macrophages in the presence of BLM in a transwell system (Fig. 5D). Real-time PCR analysis demonstrated that BLM treatment increased the expression of *Ccl2* mRNA in fibroblasts (Fig. 5E) and macrophages (Fig. 5F), which was inhibited by MSCs-IT. Immunofluorescence analysis also demonstrated a decrease in CCL2 protein levels in the MSC-IT co-culture group in both fibroblasts and macrophages (Fig. 5G, H). These data indicate that MSCs-IT inhibit the recruitment of monocytes by reducing CCL2 production from fibroblasts and macrophages, thus affecting the pathogenesis of BLM-induced SSc (Fig. 6).

## Discussion

MSC transplantation is emerging as a potential therapeutic option for SSc [25]. MSC treatment inhibits immune cell accumulation in the skin, such as neutrophils, macrophages, and T cells [18, 26]. However, how MSCs influence immune cells during SSc is still largely unknown. In this study, MSCs-IT, which possess more potent immunoregulatory capability, were used to investigate the effect of MSCs on BLM-induced SSc. We found that macrophages were the predominant immune cell population involved in the pathogenesis of BLM-induced SSc and were acted on by infused MSCs-IT. Further analysis showed that MSC-IT treatment mainly reduced CD64^+^Ly6C^+^ maturing macrophages with high CCR2 expression by inhibiting the generation of CCL2 from fibroblasts and macrophages (Fig. 6). These results indicate that MSCs-IT may target the CCL2-CCR2 axis to exert their therapeutic efficacy.

Macrophages are the most abundant immune cell population in the skin. Some studies have documented the infiltration of macrophages in skin lesions and an increase in the peripheral circulation of SSc patients, indicating their critical roles [3, 5]. The profibrotic activation of SSc macrophages has also been reported [10]. However, how macrophages exert their profibrotic function during SSc is not clear. We found that macrophages substantially infiltrated the dermis and produced a great deal of TGF-β1. These results indicate that macrophages promote the development of SSc probably by secreting the profibrotic factor TGF-β1.

Macrophages are highly heterogeneous [27]. We found that the major subpopulation influenced by MSCs-IT was CD64^+^Ly6C^+^ maturing macrophages. These macrophages expressed more CCR2 than CD64^+^Ly6C^-^ matured macrophages, indicating that these macrophages originated from peripheral CCR2^+^ monocytes. We further demonstrated that inhibiting monocyte recruitment by CCR2 blockade significantly alleviated SSc. Furthermore, we found a significant increase of CCL2 in fibrotic skin, suggesting the active recruitment of monocytes from the periphery to skin lesions. CCL2 was also found to be significantly elevated in the serum of SSc patients and correlated with the extent of skin fibrosis [28, 29]. The importance of the CCL2-CCR2 axis has been addressed in a hypochlorite-induced dermal fibrosis model. Depletion of CD11b^+^Ly6C^hi^ monocytes by anti-CD11b and anti-Ly6C antibodies as well as CCR2/CCL2 inhibition by a specific L-RNA aptamer both mitigate disease parameters [30]. In addition to the interaction between CCL2 and CCR2, CCL2 can also directly promote collagen synthesis in fibroblasts [31]. These data indicate that intervention strategies targeting CCL2 or CCR2 may achieve promising results for SSc clinical therapy.

MSCs inhibit the infiltration of macrophages in a TNF-stimulated gene 6 (TSG-6)-dependent manner [32, 33]. However, whether MSCs can regulate the expression of CCL2 in local tissues is still not clear. We found that CCL2 was mainly produced by fibroblasts and macrophages in fibrotic skin. MSCs-IT could significantly inhibit the production of CCL2 in a co-culture system, indicating that MSCs can directly modulate the CCL2-CCR2 axis. This non-contact co-culture system suggests that the secretome of MSCs can be used in SSc therapy. Moreover, the exact mechanism by which MSCs affect the expression of CCL2 should be further investigated.

In summary, our results demonstrate that MSCs-IT reduce the accumulation of macrophages by inhibiting the generation of CCL2 from fibroblasts and macrophages, thus alleviating BLM-induced SSc. This study provides insight into the mechanisms underlying MSC efficacies in autoimmune diseases.

## Materials and methods

### Isolation and culture of MSCs

MSCs were isolated from human umbilical cords as previously described [21]. Umbilical cords were obtained from healthy and full-term deliveries with parental consent following the rules set by the Ethics Committee of Soochow University. Gelatinous tissues of umbilical cords were cut into pieces after blood vessels were removed and cultured in Dulbecco’s modified Eagle’s medium (DMEM) (HyClone, Neb, USA) containing 10% FBS (Gibco, MA, USA) and 1% penicillin and streptomycin (Thermo Fisher Scientific, MA, USA) for 24 h. Then, the adherent cells were allowed to expand after the removal of the non-adherent cells.

In addition, when cultured MSCs were used for therapeutic experiments, MSCs were stimulated with 10 ng/mL IFN-γ (NBP2-34992, NOVUS, CO, USA) and 10 ng/mL TNF-α (NBP2-35076, NOVUS) for 24 h. Then, MSCs were obtained after washing three times with Phosphate Buffered Saline (PBS) to remove the cytokines.

### Animal experiments

*Rag2*^−/−^ mice and CD11b-DTR mice were obtained from the Jackson Laboratory (ME, USA), wild-type (WT) BALB/c female mice (6–8 weeks) were purchased from Shanghai SLAC Laboratory Animal Co. Ltd. (Shanghai, China) and housed in a specific pathogen-free facility. Animal care was in full compliance with the Guide for the Care and Use of Laboratory Animals, and the experimental protocols were approved by the Institutional Animal Care and Use Committee of Soochow University.

For animal experiments involving the establishment of SSc model, mice were randomly grouped according to a random number table. Each group included 4–7 mice. The sample size was determined based on previous studies [26, 34], pilot studies, the level of expected heterogeneity of samples, as well as the significance threshold (chosen at 0.05). Then, mice in the SSc group were administered subcutaneously (i.c.) with 100 μg BLM (M2100, Abmole, TX, USA) daily for 4 weeks. Correspondingly, mice administered 100 μL PBS were used as the control group.

In the MSC-IT treatment experiment, MSCs-IT (2 × 10^5^) were injected intravenously (i.v.) to SSc model on days 7, 14, and 21. In the experiment using CD11b-DTR mice, 5 μg/kg DT (D0564, Sigma-Aldrich, St Louis, MO, USA) was administered intraperitoneally (i.p.) into SSc model every 4 days for 4 weeks starting 3 days before model establishment. In the macrophage depletion experiment, 100 μL clodronate liposomes (clo) (5 mg/mL, Yeasen, Shanghai, China) or control liposomes (ctrl) were i.p. administered to SSc model every 4 days for 4 weeks starting from 3 days before the model establishment. In the CCR2 inhibition experiment, 10 mg/kg Bindarit (1493694-70-4, Selleck, TX, USA) dissolved in 2% DMSO/30% PEG300/68% ddH_2_O or DMSO was i.p. administered daily to SSc model for 4 weeks starting from day 0.

### Skin tissue processing

The dorsal skin of BLM injection site was collected for histology and flow cytometric analysis. For histology, skin samples were fixed with 4% paraformaldehyde for 24 h and then embedded into paraffin after dehydration. For flow cytometric analysis, the skin tissues were cut into pieces and digested for 2 h at 37°C in DMEM containing 2% FBS, 0.25% type I collagenase (17100017, Gibco, MA, USA), and 0.01% DNase I (D8071, Solarbio, Beijing, China).

### Hematoxylin-eosin (H&E) and Sirius Red staining

For H&E staining, skin tissues embedded in paraffin were cut into 5 µm thick slides and stained with hematoxylin for 5 min and eosin for 30 s after hydration. Skin thickness was defined as the length from the top of the granular layer to the junction between the dermis and subcutaneous fat. For Sirius Red staining, the hydrated slides were stained with a picrosirius red staining kit (PSR-1, ScyTek, UT, USA) according to the manufacturer’s instructions. Collagen content was defined as the distance from the top of the dermal layer to the junction between the dermis and subcutaneous fat. Data from all samples were included for analysis unless mice died of natural causes. The investigators were blinded to the group allocation during data analysis and statistics.

### Immunofluorescence analysis

For immunofluorescence staining of skin sections, the hydrated slides were subjected to antigen retrieval in Tris-EDTA, pH 9.0 (R20904, Yuanye Bio-Technology, Shanghai, China), at 96 °C for 30 min. Then, skin sections were incubated with 3% bovine serum albumin (Amresco, OH, USA) for 1 h to prevent non-specific staining before incubating with primary antibodies overnight at 4 °C. Finally, skin sections were incubated with the secondary antibodies for 1 h and Hoechst 33324 (H3570, Thermo Fisher Scientific) for 10 min at room temperature. The primary antibodies used were as follows: anti-α-smooth muscle actin (α-SMA) (ab124964, Abcam, MA, USA), anti-CD64 (MA529704, Invitrogen, MA, USA), anti-CD64 (139302, BioLegend, CA, USA), anti-TGF-β1 (ab215715, Abcam), anti-Vimentin (ab92547, Abcam), and anti-CCL2 (MA5-17040, Invitrogen).

For cell immunofluorescence staining of CCL2, cells were cultured on round coverslips and fixed with 4% paraformaldehyde for 30 min. Then, the cells were incubated with anti-CCL2 antibodies (MA5-17040, Invitrogen) overnight at 4 °C after incubation with 0.3% Triton X-100 (9036-19-5, Merck, St Louis, MO, USA) and 3% bovine serum albumin. Finally, the cells were incubated with the secondary antibodies for 1 h and Hoechst 33324 (H3570, Thermo Fisher Scientific) for 10 min at room temperature.

For TUNEL staining, the hydrated slides were stained with a Colorimetric TUNEL Apoptosis Assay Kit (C1098, Beyotime, Shanghai, China) according to the manufacturer’s instructions. For relevant histological analysis and statistics, the investigators were blinded to the group allocation, and data from all samples were included for analysis unless mice died of natural causes.

### Flow cytometry

Single skin cells prepared above were stained with cell-surface antibodies for 30 min at 4 °C. Then, cell phenotyping was performed on a Cytoflex Flow Cytometer (Beckman Coulter, CA, USA). The cell-surface antibodies used were as follows: PE/Cyanine7 anti-mouse CD45 (103114, BioLegend), PerCP/Cyanine5.5 anti-mouse CD45 (103132, BioLegend), APC/Cyanine7 anti-mouse CD11b (557657, BD Pharmingen), APC anti-mouse CD64 (139306, BioLegend), PE anti-mouse Ly6C (128007, BioLegend), FITC anti-mouse Ly6G (127606, BioLegend), Brilliant Violet 421 anti-mouse CD3 (100336, BioLegend), Brilliant Violet 605 anti-mouse CD19 (115540, BioLegend), and Brilliant Violet 421 anti-mouse CD192 (CCR2) (150605, BioLegend).

### Real-time PCR analysis

Total RNA extracted by TRIzol reagent (15596-08, Life Technologies, CA, USA) was subjected to reverse transcription using PrimeScript RT Master Mix (RR036A, Takara, Japan) according to the manufacturer’s instructions. Then, gene expression was measured using SYBR Green Master Mix (B21703, Bimake, TX, USA). The primers for *Ccl2* were as follows: forward primer TTAAAAACCTGGATCGGAACCAA, reverse primer GCATTAGCTTCAGATTTACGGGT.

### Co-culture of MSCs with fibroblasts or macrophages

To acquire skin-derived fibroblasts, the dorsal skin of SSc mice was removed and cut into pieces. Then, the skin pieces were cultured in DMEM containing 10% FBS, 1% penicillin, and streptomycin for 72 h, and the adherent cells were allowed to expand.

To acquire bone marrow-derived macrophages, bone marrow cells were flushed from the femurs and tibia of SSc mice and cultured in DMEM/F12 (SH30023.02, HyClone) with 10% FBS, 1% penicillin and streptomycin, and 10 ng/mL recombinant murine M-CSF (315-02, PeproTech, NJ, USA) for 7 days after red blood cell lysis.

The co-culture experiments were conducted using a transwell system. MSCs were cultured in transwell inserts and stimulated with 10 ng/mL IFN-γ (NBP2-34992, NOVUS) and 10 ng/mL TNF-α (NBP2-35076, NOVUS) for 24 h. Then, the inserts adhering MSCs were washed with PBS 3 times to remove cytokines and placed on the top of the multiwell plate adhering fibroblasts or macrophages in the presence of 10 μg/mL BLM (M2100, Abmole). The expression of CCL2 was examined at 24 h. All in vitro experiments were replicated at least three times.

### Statistical analysis

Statistical analyses were performed using Prism 9 (GraphPad Software). Data in this article are presented as the mean ± SEM. For comparisons between two groups, *P* values by Student’s *t* test were reported when there was no significance in the *F*-test and in accordance with a normal distribution. For comparisons among multiple groups, one-way ANOVA followed by Tukey’s multiple comparison test was performed when no significance in *F*-test. Each experiment was repeated at least two times. A *P* value lower than 0.05 was considered significant. *P < 0.05; **P < 0.01; ***P < 0.001; ns not significant.

## Data Availability

The datasets generated during and/or analyzed during the current study are available from the corresponding author on reasonable request.
